# Coordinate and redox interactions of epinephrine with ferric and ferrous iron at physiological pH

**DOI:** 10.1038/s41598-018-21940-7

**Published:** 2018-02-23

**Authors:** Jelena Korać, Dalibor M. Stanković, Marina Stanić, Danica Bajuk-Bogdanović, Milan Žižić, Jelena Bogdanović Pristov, Sanja Grgurić-Šipka, Ana Popović-Bijelić, Ivan Spasojević

**Affiliations:** 1 Department of Life Sciences, Institute for Multidisciplinary Research, University of Belgrade, Kneza Višeslava 1, 11030 Belgrade, Serbia; 2 The Vinča Institute of Nuclear Sciences, University of Belgrade, POB 522, 11001 Belgrade, Serbia; 3 Department of Analytical Chemistry, Innovation Center of the Faculty of Chemistry, University of Belgrade, Studentski trg 12-16, Belgrade, 11000 Serbia; 4 Faculty of Physical Chemistry, University of Belgrade, Studentski trg 12-16, 11158 Belgrade, Serbia; 5 Faculty of Chemistry, University of Belgrade, Studentski trg 12-16, 11000 Belgrade, Serbia; 6 EPR Laboratory, Faculty of Physical Chemistry, University of Belgrade, Studentski trg 12-16, 11158 Belgrade, Serbia

## Abstract

Coordinate and redox interactions of epinephrine (Epi) with iron at physiological pH are essential for understanding two very different phenomena – the detrimental effects of chronic stress on the cardiovascular system and the cross-linking of catecholamine-rich biopolymers and frameworks. Here we show that Epi and Fe^3+^ form stable high-spin complexes in the 1:1 or 3:1 stoichiometry, depending on the Epi/Fe^3+^ concentration ratio (low or high). Oxygen atoms on the catechol ring represent the sites of coordinate bond formation within physiologically relevant bidentate 1:1 complex. Redox properties of Epi are slightly impacted by Fe^3+^. On the other hand, Epi and Fe^2+^ form a complex that acts as a strong reducing agent, which leads to the production of hydrogen peroxide via O_2_ reduction, and to a facilitated formation of the Epi–Fe^3+^ complexes. Epi is not oxidized in this process, *i.e*. Fe^2+^ is not an electron shuttle, but the electron donor. Epi-catalyzed oxidation of Fe^2+^ represents a plausible chemical basis of stress-related damage to heart cells. In addition, our results support the previous findings on the interactions of catecholamine moieties in polymers with iron and provide a novel strategy for improving the efficiency of cross-linking.

## Introduction

Transient high levels of epinephrine (Epi; or adrenaline) in the bloodstream have been long recognized as the cause of cardiovascular problems that develop under chronic exposure to stress^1,2^. A number of studies have found a connection between Epi, oxidative damage, and cardiotoxicity, that is irrespective of stimulation of adrenergic receptors^3–8^. However, the chemical basis of Epi-induced oxidation under physiological conditions is not clear. Two main mechanisms have been proposed: the autooxidation of Epi and the redox interactions of Epi with iron^9–11^. The redox potential of semiquinone/Epi couple at pH = 7 is much higher compared to O_2_/superoxide radical anion (O_2_^•−^)^12^. According to this, Epi cannot directly reduce O_2_ at physiological pH. On the other hand, deprotonated Epi is susceptible to autooxidation, but this is only relevant at high pH since pKa_1_ for Epi is ∼8.6^13^. Pertinent to the present study, there is a lot of controversy regarding the redox and coordinate interactions of Epi with iron, which has two common redox states (III and II) and a capacity to form up to six coordinate bonds. Although it has previously been reported that Fe^3+^ does not oxidize Epi at physiological pH^14^, such reaction is often re-proposed^15–19^. It has been shown that catechols bind Fe^3+^ and decrease the redox potential of the Fe^3+^/Fe^2+^ couple^20–24^. However, the nature of the catechol ring substituent affects coordination ability and redox properties due to electronic, inductive, steric, and hydrophobic effects^12^. Therefore, interactions of Epi with iron have to be addressed separately and carefully put into the context of available data on catechols. Also, the interactions have to be examined under physiological pH (7.4 in human plasma), since the solubility of Fe^3+^ and the redox stability of Fe^2+^ largely depend on pH. It is important to note that the interest in the interactions of catecholamines with iron has been revived by the development of adhesive catecholamine (DOPA)-rich biopolymers and certain metal-organic frameworks. The cross-linking of catecholamine moieties in these materials depends on coordinate bonds with Fe^3+^ at pH >7^25–29^.

This study reports details on coordinate and redox interactions of Epi with Fe^3+^ and Fe^2+^ at different [Epi]/[Fe] concentration ratios and pH 7.4. UV/Vis spectrophotometry, low-T electron paramagnetic resonance spectroscopy (EPR), Raman spectroscopy, cyclic voltammetry, and oximetry were employed to study the stoichiometry, kinetics of formation, structure, and the redox potential of the Epi–Fe complexes. The stability of Epi was monitored by high performance liquid chromatography (HPLC). The study (except Raman spectroscopy and reference UV/Vis and cyclic voltammetry experiments) was performed in tris(hydroxymethyl)aminomethane (Tris) buffer, not in the typically used phosphate buffer, since phosphates bind Fe^3+^ and promote Fe^2+^ oxidation^30^, and therefore might hinder the examination of Epi-iron interactions.

## Results

### Structure of Epi–Fe^3+^ complexes

**Figure 1:**
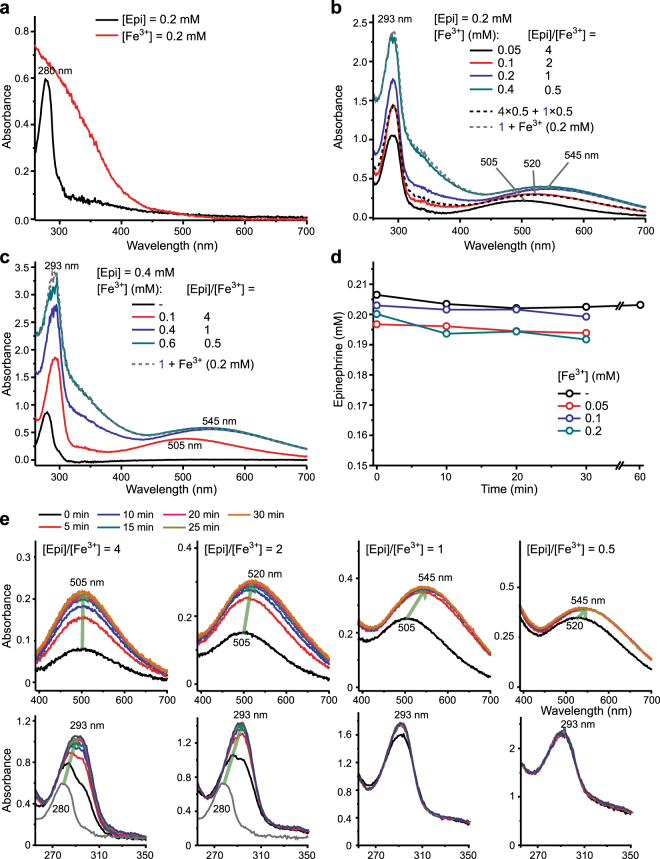
UV/Vis spectra of Epi and ferric iron in 10 mM Tris buffer, pH 7.4. (**a**) 0.2 mM Epi and 0.2 mM Fe^3+^. (**b**) 0.2 mM Epi in the presence of 0.05, 0.1, 0.2, or 0.4 mM Fe^3+^ (30 min incubation). Dashed lines represent sums of experimental spectra for different [Epi]/[Fe^3+^] = 4 and [Epi]/[Fe^3+^] = 1 (divided by 2; dark); and for [Epi]/[Fe^3+^] = 1 and free [Fe^3+^] = 0.2 mM (pale). (**c**) 0.4 mM Epi in the presence of 0.1, 0.4 or 0.6 mM Fe^3+^ (30 min incubation). The dashed lines represent the sum of experimental spectra for [Epi]/[Fe^3+^] = 1 and free [Fe^3+^] = 0.2 mM. (**d**) Stability of Epi in the presence of Fe^3+^, measured by HPLC. (**e**) Changes in UV/Vis spectra for different [Epi]/[Fe^3+^] ratios, during a 30 min incubation period. In all systems [Epi] = 0.2 mM. For clarity, the ranges 400–700 nm and 260–350 nm (gray line represents absorption from Epi only) are shown separately.

No autooxidation of Epi was observed at pH 7.4. A characteristic spectrum of Epi (λ_max_ = 280 nm) remained unaltered for at least 1 h (Fig. 1a). New bands emerged at longer wavelengths upon incubation with Fe^3+^ (Fig. 1b). These were attributed to the coloured Epi–Fe^3+^ complexes. When Fe^3+^ forms coordinate bonds, electrons in d-orbital split into high and low energy orbitals. For many ligands, including catechols, the energy difference corresponds to the wavelengths in the visible range^20^. Several [Epi]/[Fe^3+^] were studied to evaluate the stoichiometry of the complexes. [Epi] was kept constant at 0.2 mM. A broad band at λ_max_ = 505 nm was observed for [Epi]/[Fe^3+^] = 4 (Fig. 1b). The band was shifted to λ_max_ = 545 nm for [Epi]/[Fe^3+^] = 1. The absorption maximum and intensity did not change with further increase of [Fe^3+^]. The spectrum for [Epi]/[Fe^3+^] = 0.5 corresponded to the sum of experimental spectra for [Epi]/[Fe^3+^] = 1 and free [Fe^3+^] = 0.2 mM. This implies that the minimal stoichiometry is 1. Importantly, the spectrum of [Epi]/[Fe^3+^] = 2 system corresponded to the sum of spectra obtained for [Epi]/[Fe^3+^] = 4 and [Epi]/[Fe^3+^] = 1. Similar results were obtained in analogous systems with [Epi] = 0.4 mM (Fig. 1c). The 545 nm absorbance in [Epi]/[Fe^3+^] = 1 systems was 2× higher for [Epi] = 0.4 mM than [Epi] = 0.2 mM (Fig. 1c), implying that the same complex is formed regardless of Epi concentration. It is important to stress out that HPLC results showed that Fe^3+^ did not provoke detectable degradation of Epi (Fig. 1d). For the [Epi]/[Fe^3+^] = 4 system, the absorbance at 505 nm showed a gradual increase over a period of 15 min (Fig. 1e). For lower [Epi]/[Fe^3+^] ratios, the 505 nm band was replaced/shifted within 5 min to either 520 nm or 545 nm band.

**Figure 2:**
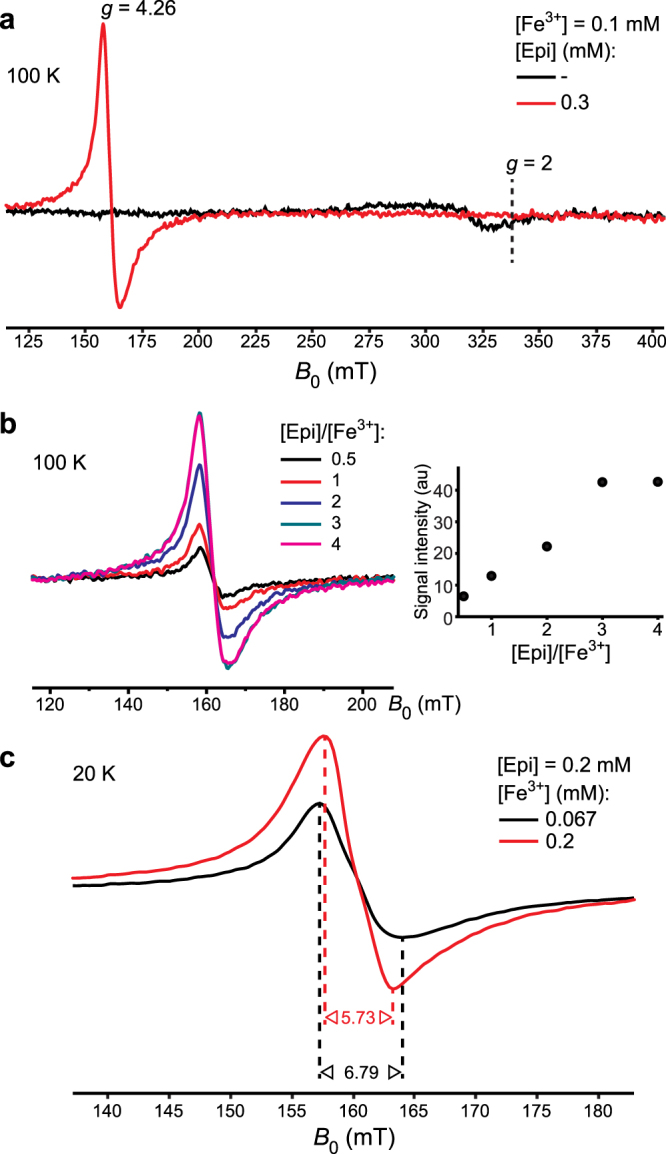
Low-T EPR spectra of Fe^3+^ in 10 mM Tris buffer, pH 7.4. (**a**) 100 K EPR spectra of Fe^3+^ in the absence or presence of Epi. (**b**) 100 K EPR spectra (left) and the intensity of the *g* = 4.26 Fe^3+^ signal (right) for different [Epi]/[Fe^3+^]. [Fe^3+^] = 0.1 mM in all samples. (**c**) 20 K EPR spectra of 0.067 mM and 0.2 mM Fe^3+^ in the presence of 0.2 mM Epi. Line-widths are given in mT. All samples were frozen after 15 min incubation at 293 K.

Using low-T EPR, it is possible to determine the total spin quantum number of Fe^3+^ in Epi–Fe complexes^31^. The 100 K EPR spectrum of 0.1 mM Fe^3+^ in 10 mM Tris buffer showed only a weak signal of low-spin Fe^3+^ (*S* = 1/2) at *g* ~ 2 (Fig. 2a)^32^. In the presence of Epi, a strong *g* = 4.26 signal that arises from high-spin Fe^3+^ (*S* = 5/2) in orthorhombic symmetry was observed. Next, [Fe^3+^] was kept constant whereas [Epi] was altered to determine the maximal number of Epi ligands per Fe^3+^. A double-integral of the high-spin Fe^3+^ signal increased with increasing [Epi], reaching the maximal value at [Epi]/[Fe^3+^] = 3 (Fig. 2b). This implies that the maximal stoichiometry is 3. Fe^3+^ remained in the high-spin state at all concentration ratios. At 100 K, the line-width of *g* = 4.26 signal was ∼7.4 mT for all investigated ratios. To gain more information about the symmetry of complexes, the spectra were acquired at 20 K (Fig. 2c), since the homogenously broadened line-width is a function of T. The line-width for 0.067 mM Fe^3+^ ([Epi]/[Fe^3+^] = 3) was broader by 1.1 mT compared to that for 0.2 mM Fe^3+^ ([Epi]/[Fe^3+^] = 1). Moreover, the signal intensity normalized to concentration was only 1.9× stronger for [Epi]/[Fe^3+^] = 1 than [Epi]/[Fe^3+^] = 3. This implies that the symmetries of the complexes formed at [Epi]/[Fe^3+^] = 3 and [Epi]/[Fe^3+^] = 1 are different, and that the former complex shows higher anisotropy. Our results are in accordance with a previous low-T EPR study of interactions of catecholamine-rich peptides with ferric iron^26^.

**Figure 3:**
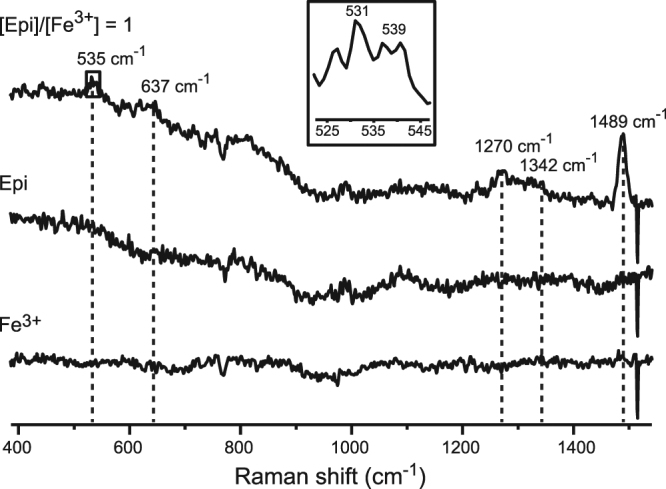
Raman spectra of 0.2 mM Epi with or without 0.2 mM Fe^3+^ in 10 mM phosphate buffer, pH 7.4. The spectrum of 0.2 mM Fe^3+^ is shown for comparison. Spectra were obtained after 15 min incubation period, using the λ = 532 nm laser excitation line. Inset: Two bands contributing to the signal at ∼535 cm^-1^.

Raman spectroscopy was conducted in phosphate instead of Tris buffer, because amides show Raman bands that are in the range of interest here. As a reference, the formation of complexes in the phosphate buffer was investigated also using UV/Vis spectroscopy (Supplementary Fig. S1). Raman spectra of the Epi–Fe^3+^ complex showed bands at ~535, 637, 1270, 1342 and 1489 cm^−1^ (Fig. 3). Band positions corresponded to the previously reported Raman spectra for Fe^3+^-catecholamine-based biopolymers and metal-organic frameworks^28,33^. These bands were (almost) negligible in the absence of Fe^3+^. It has been shown previously that the interactions of catecholamine moieties in biopolymers with Fe^3+^ drastically increase the amplitude of Raman bands^25,33^. The appearance of bands in the presence of Fe^3+^ is most likely related to the fact that Raman laser wavelength (532 nm) was close to the electronic transition in Epi–Fe^3+^ complexes (λ_max_ = 545 nm), and far from Epi absorption (λ_max_ = 280 nm). The signal at 1489 cm^−1^ showed the most prominent rise in the presence of Fe^3+^. The band has been assigned to the catechol ring vibration^28,33^. Other bands were assigned as follows: 1342 cm^−1^, C-H bending; 1270 cm^−1^, C-O stretching; 637 cm^−1^, Fe-O stretching; ~535 cm^−1^, bending/stretching of the complex^25,33^. It appears that the signal at ∼535 cm^−1^ was composed of two bands. This is the result of the binding of Fe^3+^ to two slightly different O atoms within a bidentate complex with catechol ring^27,34^. The 637 cm^−1^ band most likely reflects the bending of catechol ring away from co-planarity with the O-Fe^3+^-O that has been observed previously via crystallography^35^. The appearance of bands centred at 1270 and 1342 cm^−1^ in the presence of Fe^3+^ further corroborates Fe-O binding^28^.

### Redox properties of Epi–Fe^3+^ complexes

**Figure 4:**
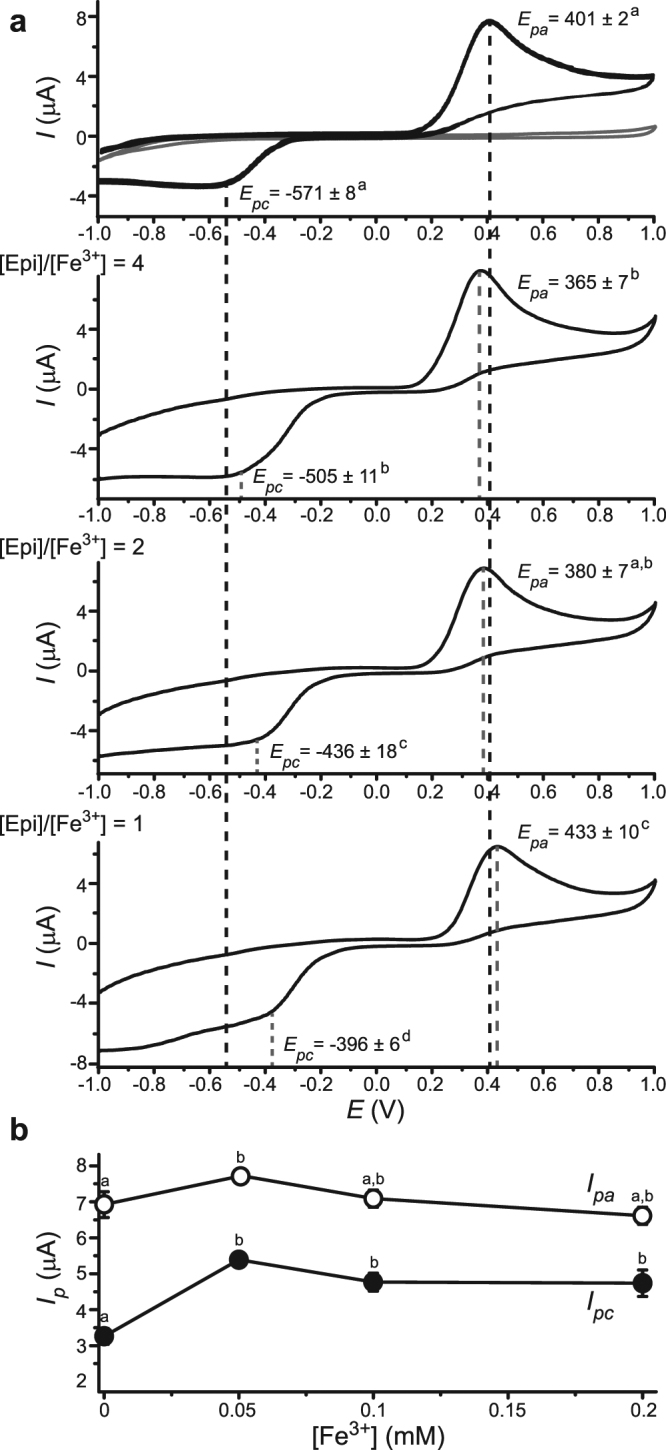
Cyclic voltammograms of 0.2 mM Epi in 10 mM Tris buffer, pH 7.4, containing different Fe^3+^ concentrations, at the boron doped diamond electrode. (**a**) From top to bottom: Epi (dark lines) and Fe^3+^ (0.2 mM; pale line), and [Epi]/[Fe^3+^] = 4, 2, and 1. The positions of oxidation/anodic (*E*_*pa*_) and reduction/cathodic (*E*_*pc*_) potentials are marked with dotted lines (dark – iron-free system; pale – all other settings). *E*_*pa*_ and *E*_*pc*_ are presented as mean values ± SE (mV). (**b**) Mean values (±SE) of anodic (*I*_pa_; open circles) and cathodic (*I*_pc_; closed circles) peak currents in CV of Epi with different [Fe^3+^]. Scan rate was 0.1 V/s. *E*_*pa*_ and *E*_*pc*_, and *I*_*pa*_ and *I*_*pc*_ not sharing a common letter were significantly different (P < 0.05).

Redox activity of Epi/Fe^3+^ systems in cyclic voltammograms (CV) was ligand-centred (Fig. 4a). CV of Fe^3+^ did not show distinctive peaks. This is most probably related to the predominance of the amorphous Fe^3+^ complex with OH^−^ ions at physiological pH (Supplementary Fig. S2). The peaks correspond to Epi oxidation (*E*_pa_ ∼ 400 mV), and to the following reduction of oxidation product(s) (*E*_pc_ ∼ −570 mV)^36^. Oxidation and reduction peak current ratios (*I*_*pa*_/*I*_*pc*_) were substantially higher than 1 (Fig. 4b), which means that the electron transfer was irreversible. This can be attributed to instability and polymerization of products of Epi oxidation^37^. Therefore we focused on anodic current. At [Epi]/[Fe^3+^] = 4 and [Epi]/[Fe^3+^] = 1, *E*_*pa*_ became more negative and positive than free Epi. At [Epi]/[Fe^3+^] = 2, *E*_*pa*_ and *I*_*pa*_ were between values for the two other ratios, which is in agreement with the UV/Vis results. Similar distribution of *E*_*pa*_ values for Epi and different [Epi]/[Fe^3+^] was observed at both slower and faster scan rates (Supplementary Fig. S3). A direct linear relationship between *I*_*pa*_*, I*_*pc*_, and the square root of scan rate implies that the currents mainly depend on two parameters: the rate at which redox species diffuse to electrode surface (*D*), and the rate constant of electron transfer (*k*_*s*_). Other interactions, such as adsorption, were negligible^38^. *D* and *k*_*s*_ were calculated using Randles–Sevick equation and Nicholson Shain method (Supplementary Fig. S3)^39,40^. For [Epi]/[Fe^3+^] = 4, the diffusion to anode was faster, whereas for [Epi]/[Fe^3+^] = 1, it was slower than Epi in the absence of iron. This can be attributed to the formation of different Epi complexes. Electron transfer from Epi to anode was particularly promoted for [Epi]/[Fe^3+^] = 1 (Supplementary Fig. S3). This may be related to the delocalization of aromatic π electrons by Fe^3+^. As a reference, Epi and Fe^3+^ were also investigated in phosphate buffer (Supplementary Fig. S4). The same complex predominated at both high and low [Epi]/[Fe^3+^], which is in agreement with UV/Vis results in this buffer. Epi in the complex showed lower *E*_*pa*_ than free Epi.

### Interactions of Epi with Fe^2+^

**Figure 5:**
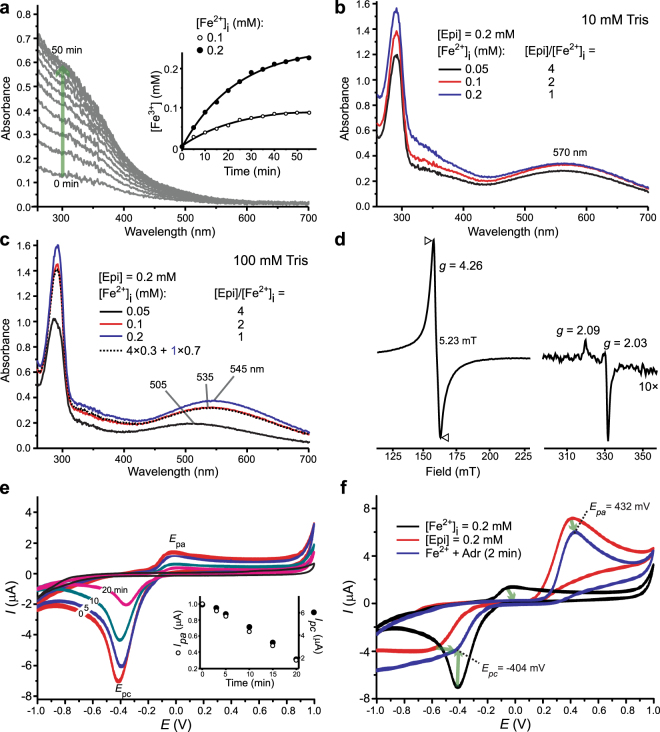
Redox interactions of 0.2 mM Epi with Fe^2+^ at pH 7.4. (**a**) UV/Vis spectra showing the oxidation of 0.2 mM Fe^2+^ to Fe^3+^ in 10 mM Tris. Inset: The accumulation of Fe^3+^ during spontaneous oxidation of 0.1 and 0.2 mM Fe^2+^; [Fe^3+^] was calculated using the absorbance at 300 nm and the FeCl_3_ calibration curve. Exponential fits are presented (*R*^2^ > 0.990). (**b**) UV/Vis spectra of Epi/Fe^2+^ systems after 1 min incubation in 10 mM Tris. (**c**) UV/Vis spectra of Epi/Fe^2+^ systems after 1 min incubation in 100 mM Tris. Dashed line represents the sum of experimental spectra. (**d**) 20 K EPR spectrum of the [Epi]/[Fe^2+^]_i_ = 2 system in 10 mM Tris after 1 min of incubation. The high field part of the spectrum was multiplied 10× for clarity (right). (**e**) Time-dependent changes of CV and peak currents (*I*_*pa*_ and *I*_*pc*_) of [Fe^2+^]_i_ = 0.2 mM in 10 mM Tris at boron doped diamond electrode. Black line – CV of Fe^3+^ (0.2 mM). (**f**) Changes (marked with arrows) of anodic and cathodic *E* and *I* of Fe^2+^ and Epi in the [Epi]/[Fe^2+^]_i_ = 2 system.

The oxidation of Fe^2+^ to Fe^3+^ at pH 7.4 was drastically promoted by Epi (Fig. 5a,b). A broad band at λ_max_ = 570 nm emerged within 1 min for different initial Fe^2+^ concentrations ([Fe^2+^]_i_). The 570 nm band has been observed previously in similar Epi–Fe^2+^ systems, and has been attributed to the Epi–Fe^3+^ complexes that are formed following Fe^2+^ oxidation^41,42^. However, an evident shift of the absorption maximum compared to Epi/Fe^3+^ systems (Fig. 1), implies that the 570 nm band may arise from some other species. Namely, the reduction of O_2_ by Fe^2+^ gives different by-products (Supplementary Table S1), including hydroxyl radical (HO^•^), a very strong oxidant. These products caused Epi degradation, as shown by HPLC (Supplementary Fig. S5). The rate constant for the reaction Epi + HO^•^ is an order of magnitude higher than Tris + HO^•^ : 2.2 × 10^10^ M^−1^ s^−1^ *vs*. 1.1 × 10^9^ M^−1^ s^−1 ^^43,44^. Therefore, 10× higher concentration of Tris (100 mM) was applied to employ Tris as an ‘antioxidative buffer’. EPR spin-trapping measurements showed that 100 mM Tris has a significantly higher capacity to remove HO^•^ than 10 mM buffer. As expected, Fe^2+^-related Epi degradation was suppressed in 100 mM Tris, being completely prevented in systems with [Fe^2+^]_i_ ≤ 0.2 mM (Supplementary Fig. S5). Under such settings, Epi-catalyzed oxidation of Fe^2+^ gave absorption bands that are characteristic for Epi–Fe^3+^ complexes: 505 nm for high [Epi]/[Fe^2+^]_i_ and 545 nm for low [Epi]/[Fe^2+^]_i_ (Fig. 5c). The spectrum for an intermediate [Epi]/[Fe^2+^]_i_ ratio was simulated to be the sum of these two. These results confirm that the shift to 570 nm is related to Epi degradation. More importantly, this corroborates that Epi is not a direct reactant in Fe^2+^ oxidation, but acts in a catalyst-like fashion. In line with this, one Epi facilitated the oxidation of two Fe^2+^ in 100 mM Tris (Supplementary Fig. S5).

Fast oxidation of Fe^2+^ in the presence of Epi was further supported by low-T EPR and cyclic voltammetry. Fe^2+^ was ‘EPR silent’ in the experimental conditions applied here (perpendicular mode EPR). Within 1 min after the addition of Epi, a strong high-spin Fe^3+^ signal was observed (Fig. 5d). In addition, lines that are characteristic for slowly tumbling organic radical appeared in the higher field^45^, confirming that partial degradation of Epi took place in 10 mM Tris. Such signal could not be observed in the systems with Fe^3+^ (not shown). Further, *I*_pa_ and *I*_pc_ in CV of Fe^2+^ showed a slow time-dependent decay in the absence of Epi (Fig. 5e). This reflects Fe^2+^ oxidation to Fe^3+^ which is CV-inactive, as discussed earlier. In contrast, a rapid change took place in the presence of Epi (Fig. 5f). Fe^2+^-related peaks were diminished and CV acquired shape with *E*_p_ and *I*_p_ values as in the CV of analogous system with Fe^3+^ ([Epi]/[Fe^3+^] = 1; Fig. 4a).

**Figure 6:**
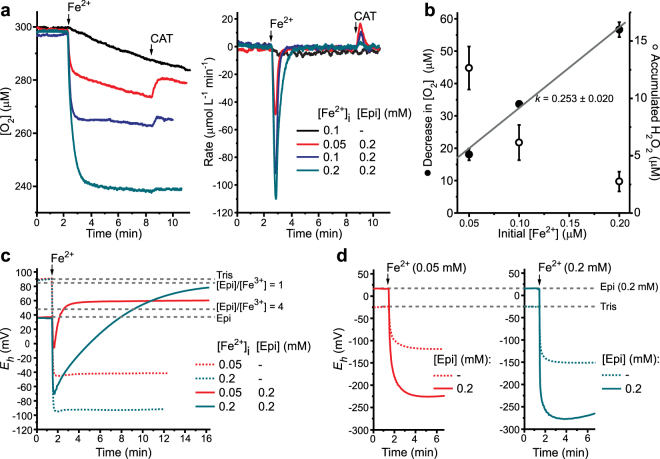
Changes in O_2_ concentration and redox potential (*E*_*h*_) in Epi/Fe^2+^ systems in 10 mM Tris, pH 7.4. (**a**) Changes of [O_2_] and rate of O_2_ consumption induced by different concentrations of Fe^2+^ in the absence or presence of 0.2 mM Epi. Top-down peaks in the right panel represent the initial rate of O_2_ consumption following the addition of Fe^2+^. (**b**) Quantification of O_2_ consumption and H_2_O_2_ accumulation, 30 s after the addition of Fe^2+^ or CAT, respectively. H_2_O_2_ accumulation was quantified by CAT-induced O_2_ release (2H_2_O_2_ → 2H_2_O + O_2_; [H_2_O_2_] = 2 × Δ[O_2_]). Data are presented as means ± SE. Closed circles represent [O_2_] and open circles represent [H_2_O_2_]. (**c**) Changes in the redox potential of 10 mM Tris buffer with or without Epi, following the addition of Fe^2+^. (**d**) Changes in the redox potential of O_2_-free 10 mM Tris buffer (under N_2(g)_) with or without Epi, following the addition of Fe^2+^. Dashed lines denote the redox potentials of referent systems (stable over time).

Next, we examined O_2_ consumption by Fe^2+^ oxidation in the presence or absence of Epi (Fig. 6a). Epi substantially increased the initial rate of O_2_ consumption. In similar experiments with Fe^3+^ no changes in [O_2_] were observed (not shown). The total decrease in [O_2_] showed a linear dependence of [Fe^2+^]_i_ with the slope *k* ∼ 0.25 (Fig. 6b). This means that four Fe^2+^ in total were being oxidized to remove one O_2_, which is in accordance with previous results on Fe^2+^ oxidation at pH 7–8^46,47^. Fe^2+^ is not ‘spent’ only on the reduction of O_2_, but also on different reactions that neither remove or produce O_2_, such as the reduction of O_2_^•−^ (generates H_2_O_2_), Fenton reaction, and the reaction with HO^•^ (Supplementary Table S1). Initial rates of O_2_ consumption and *k* value were used to calculate the initial rates of Fe^2+^ oxidation. They were as follows: ∼0.2 min^−1^ for Fe^2+^; and 3.95 ± 0.22, 3.73 ± 0.11, and 2.36 ± 0.08 min^−1^ for [Epi]/[Fe^2+^]_i_ = 4, 2, and 1, respectively. Catalase (CAT) was added 5 min following the Fe^2+^-induced drop in [O_2_] to evaluate H_2_O_2_ accumulation. At low [Fe^2+^]_i_, almost all consumed O_2_ was converted to H_2_O_2_, whereas high [Fe^2+^]_i_ prevented H_2_O_2_ accumulation (Fig. 6b). This is in line with previous studies of Fe^2+^/O_2_ system at pH ∼7 showing that H_2_O_2_ removal is promoted with increasing [Fe^2+^]_i_^46,47^. It is noteworthy that we could not detect O_2_^•−^ or HO^•^ in these systems using EPR spin-trapping, probably because Epi–Fe^2+^ complex reduced the paramagnetic spin-adducts^48^. The Epi–Fe^2+^ complex was further examined by measuring the redox potential (*E*_*h*_) under aerobic and anaerobic conditions. As a reducing agent, Fe^2+^ caused a considerable and relatively stable drop of *E*_*h*_ (Fig. 6c). In the presence of Epi, the change in *E*_*h*_ was less pronounced and partially reversible. It can be observed that *E*_*h*_ for [Epi]/[Fe^2+^]_i_ = 4 was stabilized at higher values compared to *E*_*h*_ for analogous system with Fe^3+^ (Fig. 6c). This is probably related to the accumulation of H_2_O_2_ (Fig. 6a), which is an oxidizing species and thus increases *E*_*h*_. At [Epi]/[Fe^2+^]_i_ = 1, *E*_*h*_ slowly approached a plateau at the value that was obtained for [Epi]/[Fe^3+^] = 1 system (Fig. 6c). This is in line with the absence of H_2_O_2_ accumulation (Fig. 6a). It is worth mentioning that *E*_*h*_ for [Epi]/[Fe^3+^] = 1 was higher compared to *E*_*h*_ for [Epi]/[Fe^3+^] = 4, which is in accord with the cyclic voltammetry. Fast oxidation hindered the determination of inherent redox properties of the Epi/Fe^2+^ system. Therefore, additional measurements were conducted under anaerobic conditions (Fig. 6d). The addition of Fe^2+^ provoked an irreversible decrease of *E*_*h*_ that was significantly more pronounced in the presence of Epi. Final *E*_*h*_ was more than 120 mV lower in the Epi/Fe^2+^ systems compared to *E*_*h*_ of corresponding Fe^2+^ solutions without Epi. This implies that Epi and Fe^2+^ form a strong reducing agent.

## Discussion

Epi and Fe^3+^ build high-spin complexes at pH 7.4, with 1:1 (λ_max_ at 545 nm) or 3:1 (λ_max_ = 505 nm) stoichiometry depending on the [Epi]/[Fe^3+^] concentration ratio. The 1:1 complex is bidentate. Coordinate bonds are formed with O atoms on the catechol ring (Supplementary Fig. S6). Electrochemical data showed that Fe^3+^ does not drastically affect redox properties of Epi in Tris buffer, whereas Epi in 1:1 complex in the phosphate buffer was more susceptible to oxidation than free Epi. Nevertheless, Epi was stable in the presence of Fe^3+^. This was confirmed by HPLC and UV/Vis. The formation of 3:1 complex preceded the formation of 1:1 complex at low [Epi]/[Fe^3+^]. Hence, the 505 nm band is not related to the formation of quinones or some other products. In addition, the production of adrenochrome (λ_max_ = 480 nm), a common derivative of Epi oxidation, was not observed. This appears to be in discord with some previous reports. However, those have been performed in atypical or complex settings, which might be prone to copper impurities, such as highly acidic media^15,16^, biochemical assays^17–19^, or long incubation in multi-component buffers^10^.

Epi and Fe^2+^ form a complex, most likely in 1:1 stoichiometry^41^, which represents a strong reducing agent. The oxidation of Fe^2+^ was facilitated at least 10× by Epi. A modelling study estimated that Fe^2+^ transfers 1.3 electrons to the electron-rich catechol ring^49^, which might result in destabilization of the complex. The promotion of Fe^2+^ oxidation by Epi might be further explained by the fact that ligands with harder donor sites are better Fe^3+^ stabilizers and decrease the redox potential of Fe^3+^/Fe^2+^ pair^23^. The stability constants for catechol complexes with Fe^3+^ are significantly higher than complexes with Fe^2+ ^^22^. According to Pearson’s Hard and Soft Acids and Bases principle, Fe^3+^ is hard, whereas Fe^2+^ is borderline Lewis acid. Hydroxyl groups represent hard bases^50^. It has been calculated that Highest Occupied Molecular Orbital in Epi at physiological pH is located on the catechol ring, and that electrons in the ring are redistributed towards C atoms that carry hydroxyl groups^51^. This makes these -OH groups even harder bases than hydroxyl groups on aliphatic chains. Hence, Epi binds stronger to Fe^3+^ than to Fe^2+^ due to matched hard–hard interaction. Epi-catalyzed oxidation of Fe^2+^ by O_2_ results in the production of H_2_O_2_ and HO^•^, and in the formation of Epi–Fe^3+^ complexes. Epi is not an electron donor. It is degraded only by reactive by-products, which was prevented by HO^•^-scavenging activity of high-concentration Tris.

The 1:1 complex appears to be more (patho)physiologically relevant species. Iron is the most abundant transition metal in human plasma with a total concentration of 10–30 μM. The amount of labile iron (different redox-active Fe complexes with small ligands) is variable^52^. [Epi] in human plasma may reach values >50 nM in response to stress. The concentration can be drastically higher locally, as well as in some pathological conditions, such as adrenal gland tumours (up to 3.5 μM), that are also accompanied by cardiovascular complications^53^. Nevertheless, the concentration of labile iron still appears to be higher than Epi. In addition, the 1:1 complex develops in the phosphate buffer even at higher [Epi]/[Fe^3+^]. Epi may contribute to the labile iron pool in plasma, thus increasing the solubility of iron, and promoting its redox activity, which is a foe of physiological milieu. The 1:1 complex may even act as a distinct entity with functions that are yet to be discovered. Importantly, Epi-catalyzed oxidation of Fe^2+^, the soluble form of iron in human plasma, represents a plausible chemical mechanism of the cardiotoxic effects of stress-related high Epi concentrations. Hydrogen peroxide is known to pass the cell membrane to hit sensitive intracellular targets, whereas HO^•^ induces membrane lipid peroxidation^52^. It is important to point out that Epi-induced oxidative stress requires the reduced form of iron and that Epi cannot reduce Fe^3+^. This implies that reducing agents (*i.e*. antioxidants) might not be a beneficial prophylaxis for cardiovascular diseases^54,55^.

The dependence of stoichiometry on the concentration ratio that was established here, has been observed previously for the binding of catecholamine moieties in biopolymers to Fe^3+ ^^56^. This, as well as the analogy in EPR and Raman spectra^26,28,33^, implies that Epi and Fe^3+^ might represent a good experimental model for cross-linking in catecholamine-rich polymers. Slow cross-linking reaction with Fe^3+^ is the rate-limiting step in the development of adhesion in such polymers. We have shown that Epi–Fe^3+^ complexes developed ~10× faster when Fe^2+^, instead of Fe^3+^, was available to Epi. This may be explained by the fact that the highly soluble Fe^2+^ is generally more accessible to ligands, whereas Epi competes with OH^−^ ions for Fe^3+ ^^57^. The pre-binding of catecholamine moieties to Fe^3+^ at low pH has been proposed to increase the efficiency of cross-linking that is initiated by pH increase^27^. Our results indicate that the application/pre-binding of Fe^2+^ followed by (spontaneous) oxidation at pH >7, may be a simple alternative strategy for cross-linking promotion.

## Methods

### Chemicals

All chemicals were of analytical grade: Epi (L-adrenaline; Fluka Biochemika, Buchs, Switzerland), FeCl_3_ (Analytika Ltd., Prague, Czech Republic), FeSO_4_ (Sigma-Aldrich, St. Louis, MO, USA), Tris (Serva, Heidelberg, Germany). All experiments were performed using bidistilled deionized ultrapure (18 MΩ) water. Stock solutions of Epi (0.2 or 0.4 mM) were prepared fresh each day in 10 mM Tris buffer pH 7.4 and stored on ice in the dark. For Raman spectroscopy and reference UV/Vis and cyclic voltammetry experiments Epi stock solutions were prepared in phosphate buffer (10 mM KH_2_PO_4_, pH 7.4). Epi in solution was repeatedly checked for stability using spectrophotometry. Stock solutions of FeCl_3_ (40 mM) and FeSO_4_ (40 mM) were prepared in water. Incubation and measurements were conducted in the dark at 293 K (except EPR).

### UV/VIS spectroscopy

UV-Vis absorption spectra were obtained using 2501 PC Shimadzu spectrophotometer (Kyoto, Japan). Sample volume was 1 mL. Scan time was 50 s. Samples were freshly prepared and immediately scanned at wavelengths from 800 to 200 nm. Changes of spectra were monitored for at least 30 min.

### EPR spectroscopy

Low-T EPR spectra of Fe^3+^ were recorded on a Bruker Elexsys II E540 spectrometer operating at X-band (9.4 GHz). Measurements at 100 K were performed using the Bruker N_2_ Temperature Controller ER4131VT. Measurements at 20 K were conducted using Oxford Instruments ESR900 helium cryostat. The experimental parameters were: microwave power, 3.2 mW; scan time, 80 s; modulation amplitude, 0.5 mT; modulation frequency, 100 kHz; number of accumulations, 4 (at 100 K) and 2 (at 20 K). At both T, signal amplitude *vs*. power plot was built to determine the maximum power value. Approximately one half of the maximal power was applied to avoid saturation. All spectra were baseline corrected. Samples were placed in quartz cuvettes (Wilmad-LabGlass, Vineland, NJ, USA) after 1 min (Fe^2+^) or 15 min (Fe^3+^) incubation period, and quickly frozen in cold isopentane.

EPR spin-trapping experiments were conducted using DEPMPO spin-trap (Enzo Life Sciences, Inc. Farmingdale, NY, USA) at the final concentration of 5 mM. Hydroxyl radical was generated in the Fenton reaction: Fe^2+^ (0.4 mM) + H_2_O_2_ (1.2 mM; Carlo Erba Reagents, Milano, Italy). Spectra were recorded after 5 min incubation period using a Varian E104-A EPR spectrometer operating at X-band (9.53 GHz) with the following settings: modulation amplitude, 0.2 mT; modulation frequency, 100 kHz; microwave power, 20 mW; time constant, 32 ms; scanning time, 2 min. Parameters of simulation (performed in WINEPR SimFonia; Bruker Analytische Messtechnik GmbH, Darmstadt, Germany): DEPMPO/HO, *a*_N_ = 1.40 mT, *a*_H_ = 1.32 mT, *a*_H_^γ^ = 0.03 mT (3 H), *a*_P_ = 4.73 mT; DEPMPO/C, *a*_N_ = 1.44 mT, *a*_H_ = 2.15 mT, *a*_P_ = 4.63 mT.

### Raman spectroscopy

The Raman spectra were recorded using a DXR Raman microscope(Thermo Fisher Scientific, Waltham, MA, USA). Aliquots of 5 μL solution were placed on calcium fluoride glass and measured under the microscope (with objective magnification of 50×), using the 532 nm laser excitation line, with a constant power illumination of 10 mW. The exposure time was 30 s, with 10 exposures. The laser spot diameter was 1 μm. The scattered light was analyzed by the spectrograph equipped with a 900 lines mm^−1^ grating using 50 μm slit as spectrograph aperture. In the cases with high fluorescence background, automatic fluorescence correction was performed using the OMNIC software (Thermo Fisher Scientific).

### Cyclic voltammetry

The voltammetric measurements were performed using a potentiostat/galvanostat CHI 760b (CH Instruments, Inc, Austin, TX, USA). The electrochemical cell was equipped with: a boron-doped diamond electrode (inner diameter of 3 mm; Windsor Scientific LTD, UK) embedded in a polyether ether ketone body with an inner diameter of 3 mm, a resistivity of 0.075 Ω cm, and a boron doping level of 1000 ppm (working electrode); Ag/AgCl (3 M KCl) (reference electrode); and Pt wire (counter electrode).

### Oximetry and redox potential measurements

[O_2_] was determined using a Clark type oxygen electrode (Hansatech Instruments Ltd., King’s Lynn, UK) operating with Lab Pro interface and Logger Pro 3 software (Vernier, Beaverton, OR, USA). All systems were stirred and recorded for 2–5 min before Fe^2+^ addition to establish the stability of baseline and zero rate of O_2_ change. Decrease in [O_2_] was monitored for 5 min before the addition of CAT (100 IU; Sigma-Aldrich). Redox potentials were recorded by InLab Redox Micro redox electrode operating with Seven Compact S210 pH meter and LabX software (Mettler-Toledo International Inc., Columbus, OH, USA). Measurements under anaerobic conditions were performed in N_2_ dry box (Plas-Lab, Lansing, MI, USA).

### Statistics

All experiments were performed in triplicate. Statistical analysis was performed in STATISTICA 8.0 (StatSoft Inc., Tulsa, OK, USA) using nonparametric 2-tailed Mann–Whitney test (P < 0.05) and optimal curve fitting protocols. The goodness of fits was evaluated by R^2^ (the adjusted r-square value).

## Electronic supplementary material

Supplementary Information
