# Supplementary material for: Coordinate and redox interactions of epinephrine with ferric and ferrous iron at physiological pH
Source: Sci Rep. 2018 Feb 23;8:3530. doi: 10.1038/s41598-018-21940-7 (PMC5824886; doi:10.1038/s41598-018-21940-7)
Supplement: Supplementary file 1 — Supplementary Information [file 41598_2018_21940_MOESM1_ESM.pdf]

Coordinate and redox interactions of epinephrine with ferric and ferrous iron at physiological pH

Jelena Korać, Dalibor M. Stanković, Marina Stanić, Danica Bajuk-Bogdanović, Milan Žižić, Jelena Bogdanović Pristov, Sanja Grgurić-Šipka, Ana Popović-Bijelić, Ivan Spasojević

Supplementary Information

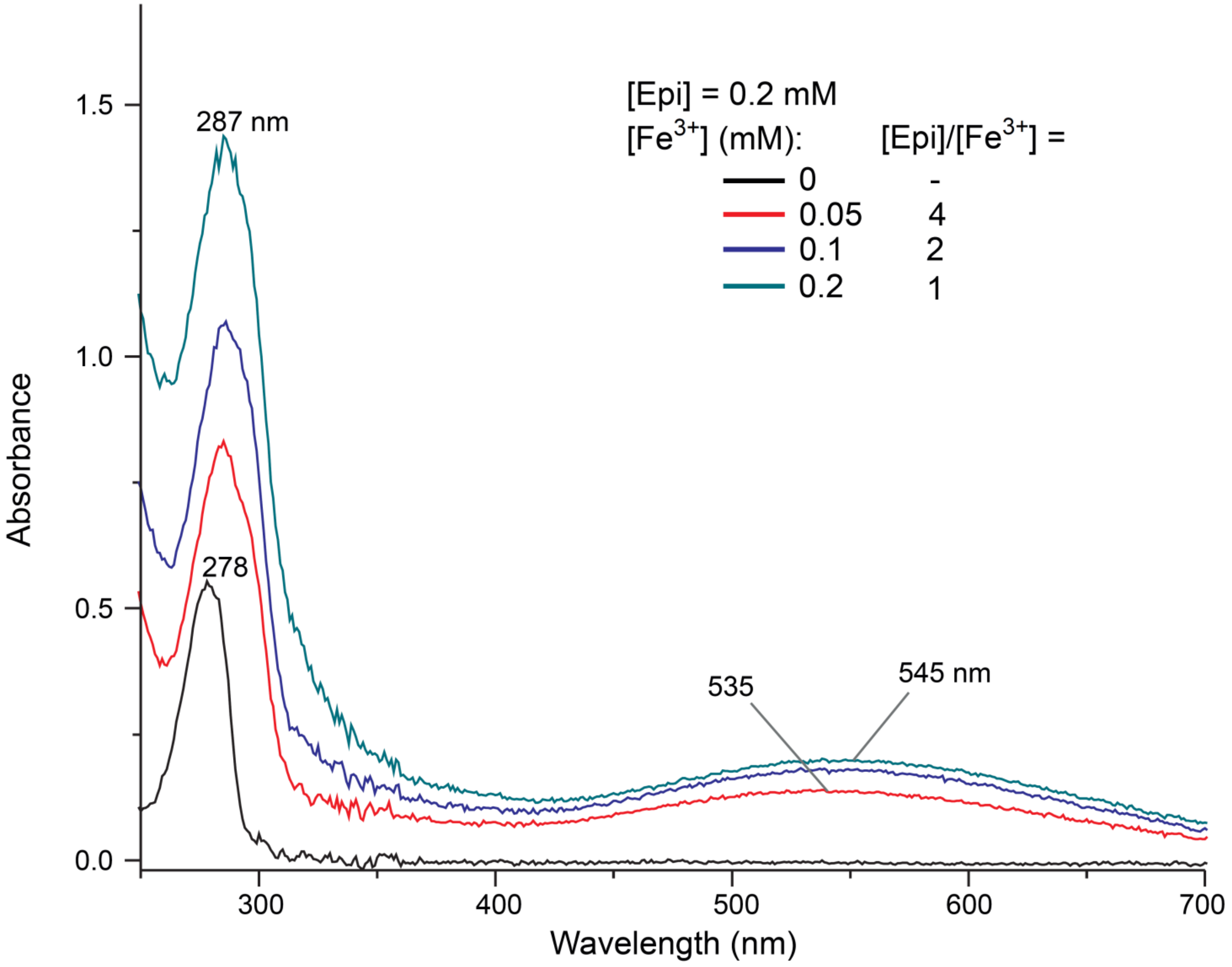

Figure S1 UV/Vis spectra of Epi and ferric iron in 10 mM phosphate buffer, pH 7.4. In contrast to Tris, the same complex appears to predominate at both low and high [Epi]/[Fe^{3+}] (λ_{max} = 545 nm), most likely in relation to the high affinity of phosphates for Fe^{3+}. The spectra were acquired following 15 min incubation, and remained unaltered for at least 1 h.

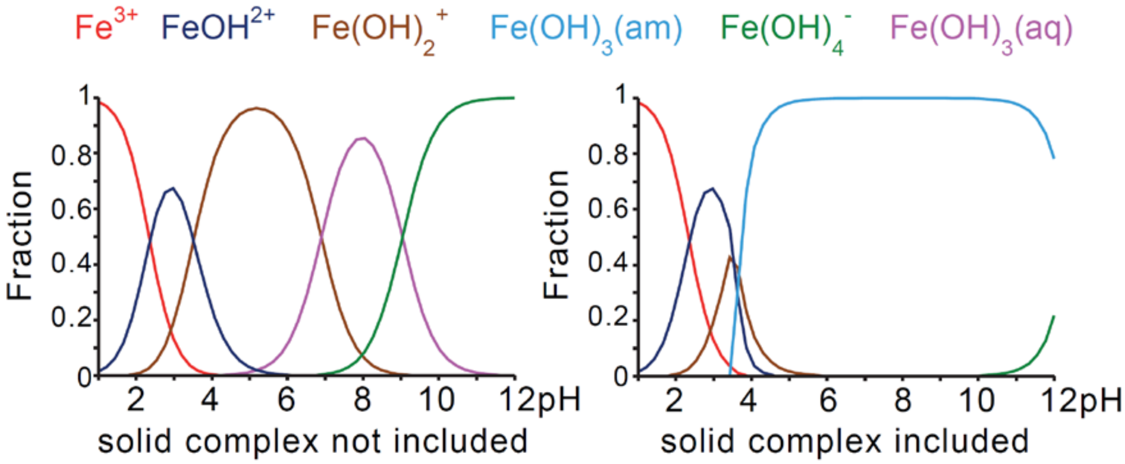

Figure S2 Speciation diagrams of Fe^{3+} in water. Diagrams were prepared in Hydra-Medusa Software, using the following parameters: [Fe^{3+}] = 0.1 mM; pH range 1–12; T = 293 K.

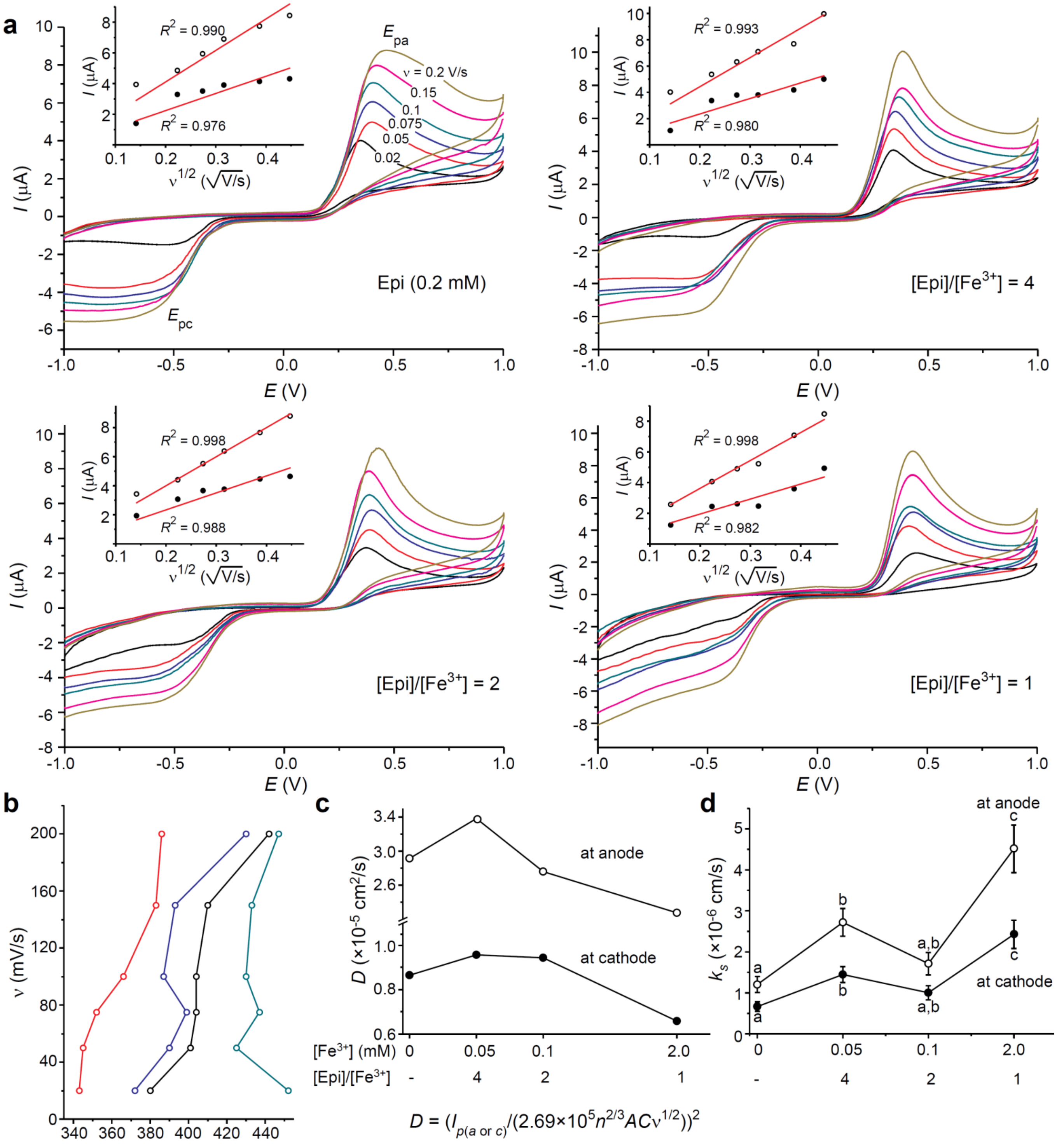

$$D = (I_{p(a\ \mathrm{or}\ c)}/(2.69\times10^5 n^{2/3} A C \nu^{1/2}))^2$$

$$k_s = \frac{10^{(0.294(nF\Delta E_p/RT - 2.218)^{-1} - 0.0803 - 0.108(nF\Delta E_p/RT - 2.218))}}{\sqrt{RT/nF\nu D}}$$

Figure S3 Scan rate analysis of the Epi/Fe^{3+} systems. (**a**) Cyclic voltammograms of Epi in absence or presence of Fe^{3+} at the boron doped diamond electrode obtained at different scan rates (v = 0.02–0.2 V/s). The inset figures in each panel represent the dependence between I_{pa} (open circles) and I_{pc} (closed circles; absolute values) and $v^{1/2}$. Linear fit and R^2 values are presented. (**b**) E_{pa} for Epi and [Epi]/[Fe^{3+}] = 4, 2,

and 1 at different *v*. (**c**) *D* for Epi and different [Epi]/[Fe^{3+}]. Randles–Sevick equation (at the bottom middle): *n*, number of transferred e^-; *A*, area of the working electrode (0.0707 cm^2); *C*, concentration of redox species in solution (mol/cm^3). (**d**) Rate constants of electron transfer (k_s) for Epi and different [Epi]/[Fe^{3+}]. Results are presented as means (± SE) of measurements made at various *v*. k_s not sharing a common letter are significantly different ($P < 0.05$). Nicholson Shain calculus (at the bottom right): *R*, standard gas constant; *T*, temperature (298 K); *F*, Faraday's constant; ΔE_p, the difference between E_{pa} and E_{pc} taken at various *v*.

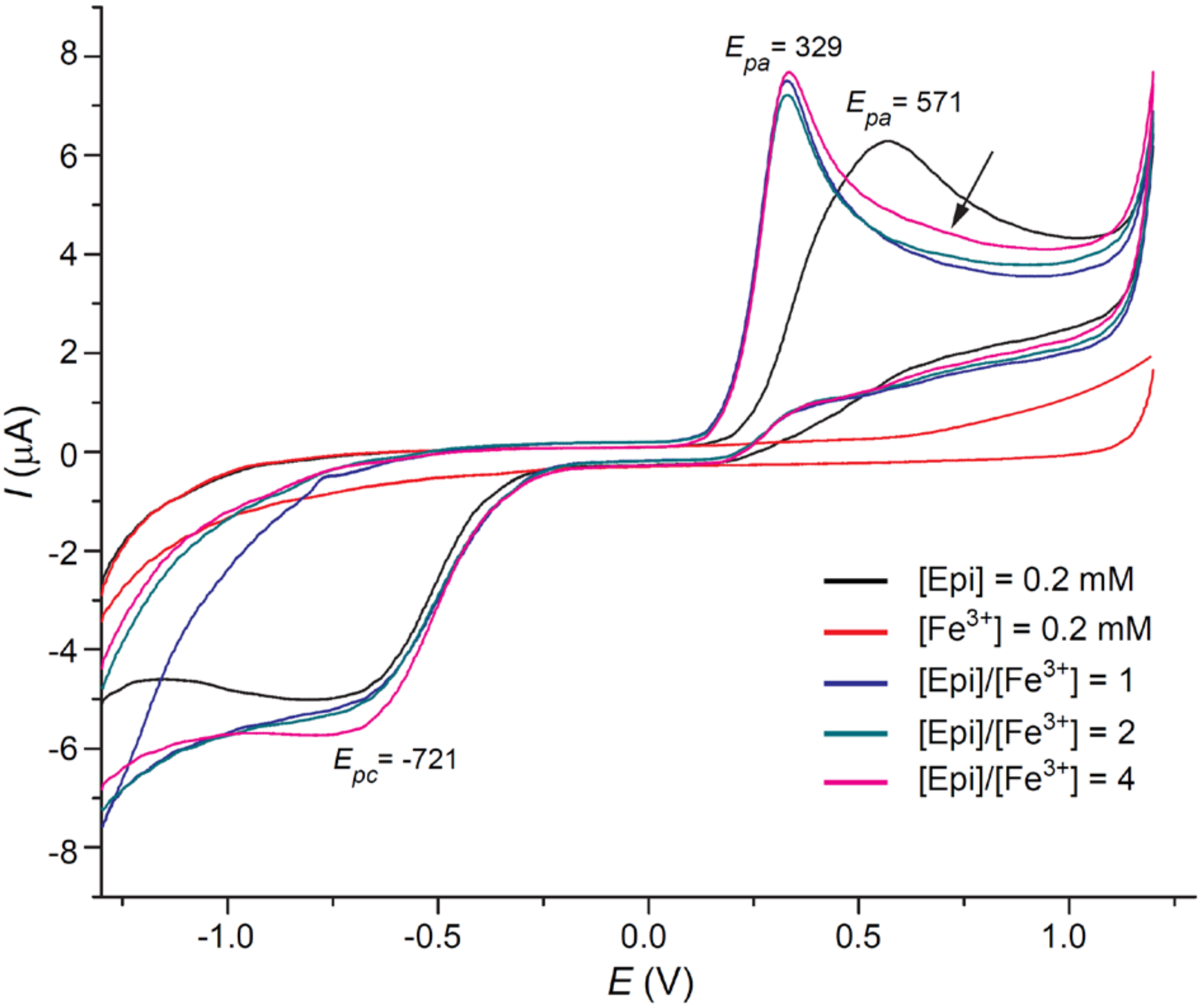

Figure S4 Cyclic voltammograms of Epi in absence or presence of Fe^{3+} in 10 mM potassium phosphate buffer, pH 7.4, at the boron doped diamond electrode. The oxidation/anodic (E_{pa}) and reduction/cathodic (E_{pc}) potentials are presented. Scan rate was 0.1 V/s.

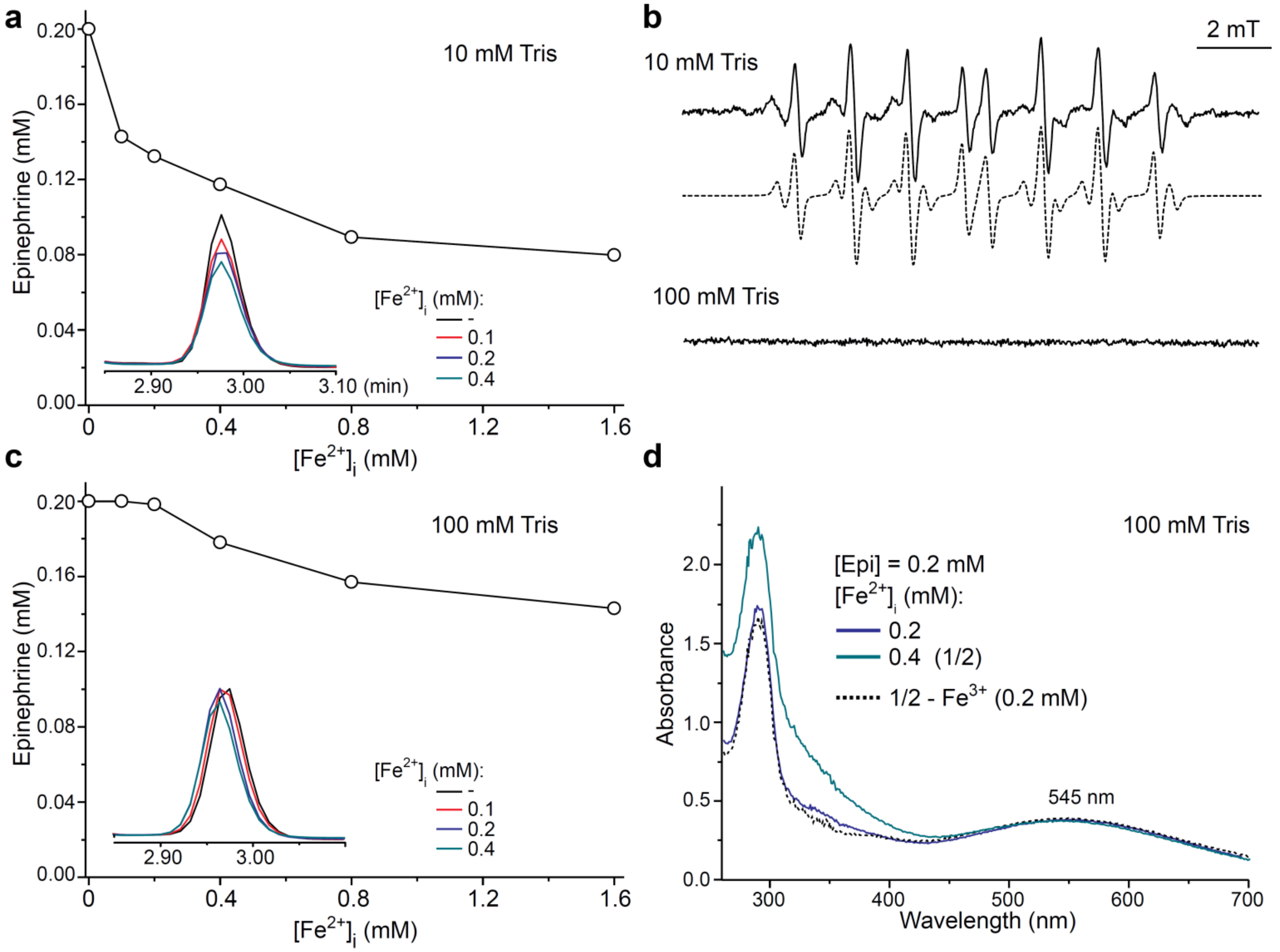

Figure S5 Antioxidative performance of 10 mM and 100 mM Tris, pH 7.4. (**a**) The concentration of Epi following 5 min incubation with different $[Fe^{2+}]_i$ in 10 mM Tris buffer. Inset: Epi peaks in HPLC chromatograms. (**b**) EPR spectra of adducts of DEPMPO spin trap (5 mM), illustrating the capacity of Tris to remove hydroxyl radical ($HO^{\bullet}$ is produced in the Fenton reaction: Fe^{2+} (0.4 mM) + H_2O_2 (1.2 mM)). Spectrum in 10 mM Tris is composed of DEPMPO adducts with $HO^{\bullet}$ (65%) and Tris-derived C-centred radical (35%), as determined by spectral simulation (dashed line). No EPR signal could be observed in 100 mM Tris. (**c**) The concentration of Epi following 5 min incubation with different $[Fe^{2+}]_i$ in 100 mM Tris buffer. Inset: Epi peaks in HPLC chromatograms.(**d**) UV-Vis spectra of Epi/Fe^{2+} systems after 5 min incubation in 100 mM Tris. No further changes were observed. Dashed line represents the subtraction of experimental spectra. The resulting spectrum with λ_{max} = 545 nm in the system with $[Epi]/[Fe^{2+}]_i = 0.5$, represents the sum of the spectrum for $[Epi]/[Fe^{2+}]_i = 1$ and the spectrum of $[Fe^{3+}]$ = 0.2 mM.

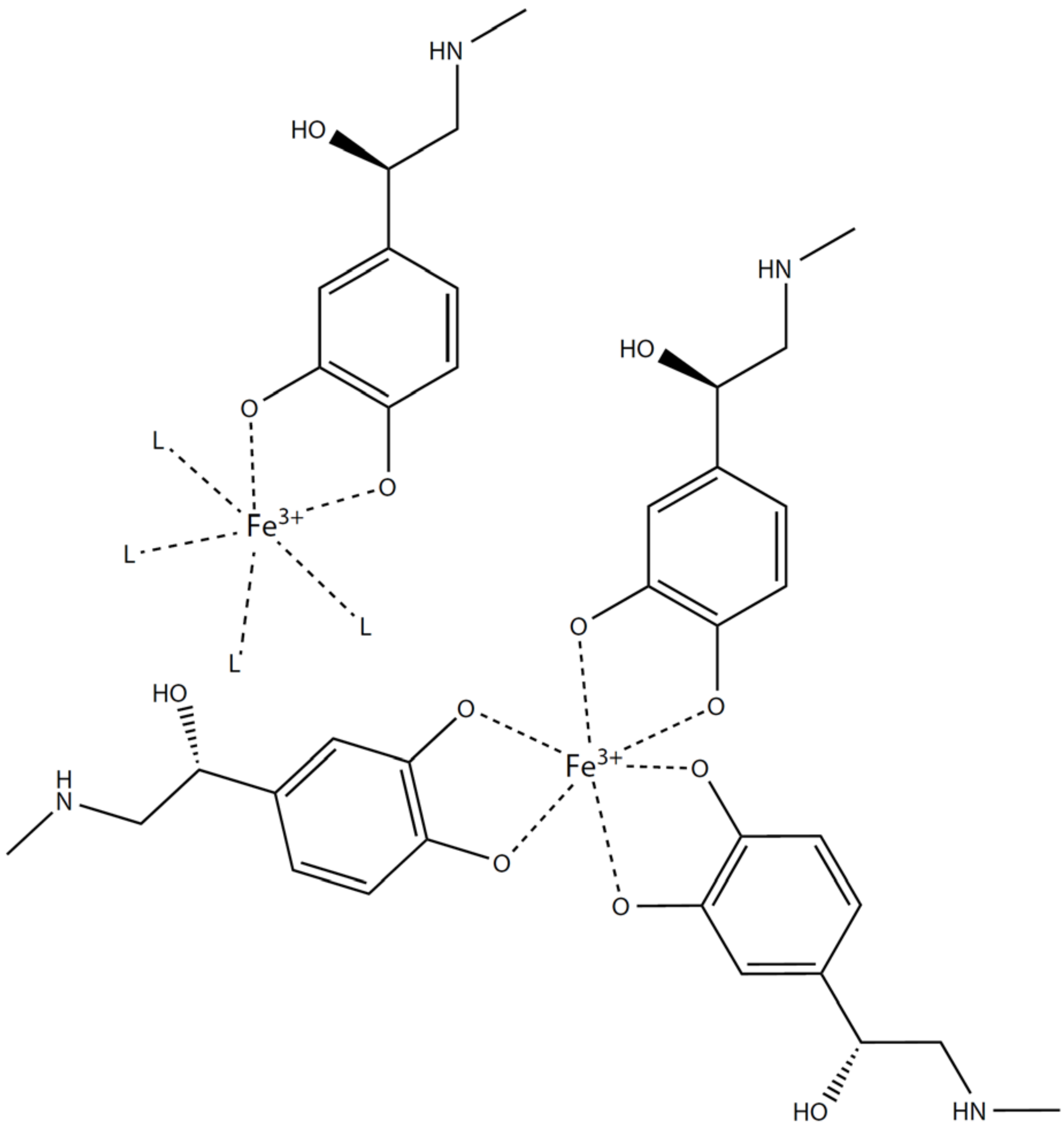

Figure S6 Schematic presentation of the chemical structures of Epi–Fe^{3+} complexes. L – other ligands (*e.g.* OH^-, HPO_4^{2-}, $H_2PO_4^-$, H_2O).

Table S1 Reactions that are relevant for Fe^{2+} oxidation at pH 7.4.

No	Reaction	k (M^{-1} s^{-1})	Ref
1	$Fe^{2+} + O_2 \rightarrow Fe^{3+} + O_2^{\bullet-}$	4×10^{-2}	(a)
2	$Fe^{2+} + O_2^{\bullet-} + 2H^+ \rightarrow Fe^{3+} + H_2O_2$	1×10^7	(b)
3	$Fe^{3+} + O_2^{\bullet-} \rightarrow Fe^{2+} + O_2$	1.5×10^8	
4*	$Fe^{2+} + H_2O_2 \rightarrow Fe^{3+} + HO^{\bullet} + OH^-$	10^2	
5	$Fe^{2+} + HO^{\bullet} \rightarrow FeOH^{2+}$	3.2×10^8	
6**	$2H_2O_2$ (CAT) $\rightarrow 2H_2O + O_2$		

* Fenton reaction; **The mechanism of catalase-mediated degradation of H_2O_2. The concentration of accumulated H_2O_2 is calculated as $2\times\Delta[O_2]$ that is induced by CAT.

(a) King, D. W.; Lounsbury, H. A.; Millero, F. J. *Environ. Sci. Technol.,* 1995, **29**, 818-825.

(b) Halliwell, B. & Gutterridge, J. M. C. Free Radicals in Biology and Medicine, 4th ed, Clarendon Press, Oxford, 2007.
